# Supplementary material for: FoxO transcription factors actuate the formative pluripotency specific gene expression programme
Source: Nat Commun. 2024 Sep 9;15:7879. doi: 10.1038/s41467-024-51794-9 (PMC11384738; doi:10.1038/s41467-024-51794-9)
Supplement: Supplementary file 1 — Supplementary Information [file 41467_2024_51794_MOESM1_ESM.pdf]

Supplementary Information

FoxO transcription factors actuate the formative pluripotency specific gene expression programme

Laura Santini[1,2], Saskia Kowald[1], Luis Miguel Cerron-Alvan[1,2], Michelle Huth[1,2], Anna Philina Fabing[1], Giovanni Sestini[2,3], Nicolas Rivron[3] and Martin Leeb[1*]

[1] Max Perutz Laboratories Vienna, University of Vienna, Vienna BioCenter, 1030 Vienna, Austria.

[2] Vienna BioCenter PhD Program, Doctoral School of the University of Vienna, Medical University of Vienna, 1030 Vienna, Austria.

[3] Institute of Molecular Biotechnology of the Austrian Academy of Sciences (IMBA), Vienna BioCenter, 1030 Vienna, Austria.

*Corresponding author, Email: martin.leeb@univie.ac.at

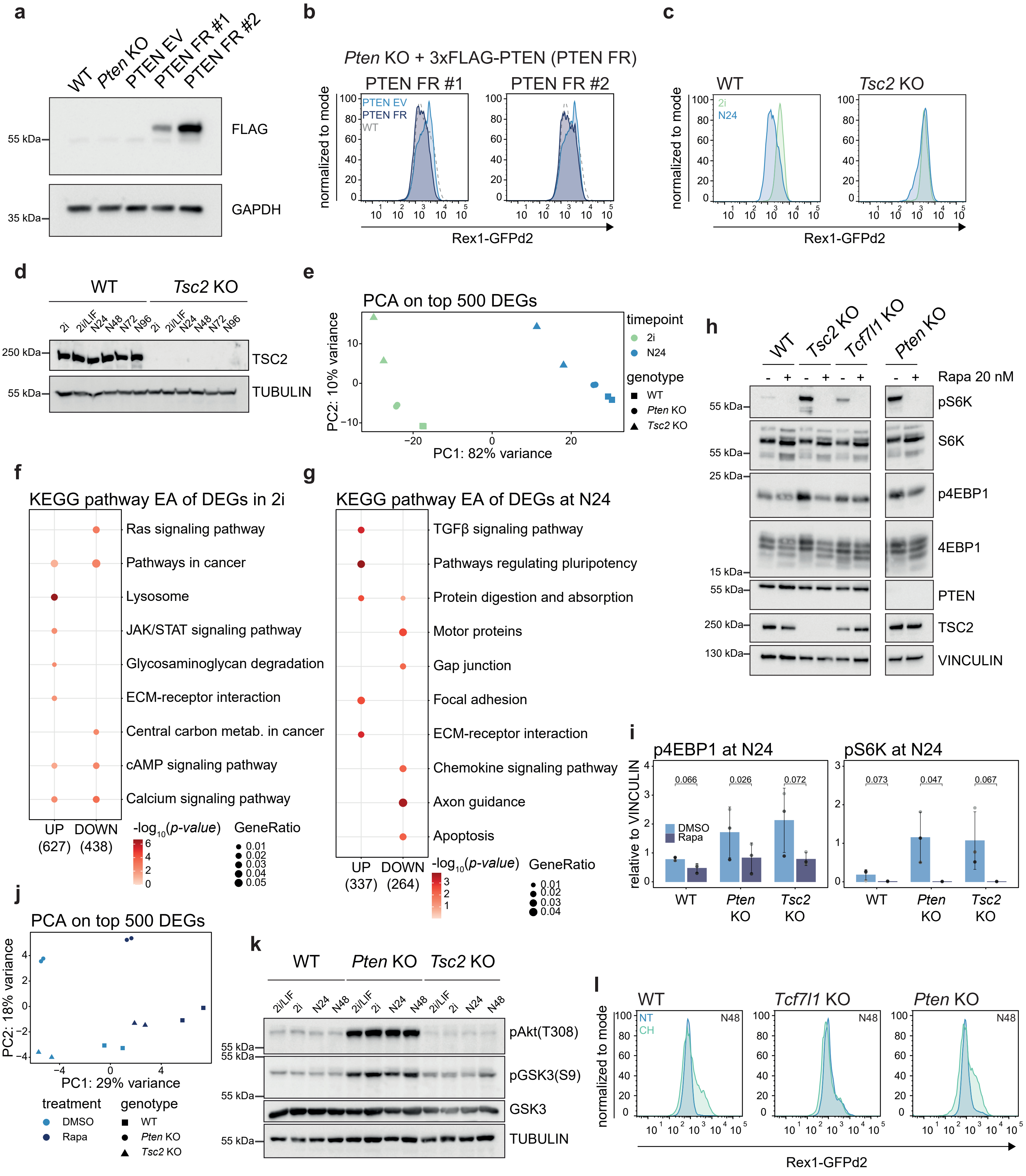

Supplementary Figure 1

Supplementary Fig. 1 | mTORC1 inhibition only partially rescues *Pten* KO phenotype.

a, Western blot for FLAG expression in indicated cell lines (FR=flag rescue, EV=empty vector). TUBULIN serves as loading control.

b, Flow cytometry analysis of Rex1-GFP levels in WT (grey dashed), PTEN EV (light blue) and PTEN FR mESCs (dark blue) at N24. Representative of n=3 independent experiments.

c, Flow cytometry analysis of Rex1-GFP levels in WT and in *Tsc2* KO cells in 2i (green) and at N24 (blue). One representative of n=10 independent experiments is shown.

d, Western blot for TSC2 expression in WT and *Tsc2* KOs in 2i, 2i/LIF, N24, N48, N72 and N96. TUBULIN serves as loading control.

e, Principal component analysis (PCA) based on top 500 DEGs in RNA-Seq data of WT, *Pten* and *Tsc2* KOs in 2i (green) and N24 (blue).

f, KEGG pathway enrichment analysis (EA) on upregulated (UP) and downregulated (DOWN) genes in *Pten* and *Tsc2* KO ESCs. Top 5 categories for each list are shown. Dot colour indicates *p* values (only $p \leq 0.1$ are shown). Dot size indicates GeneRatio (overlap size/category size).

g, Similar to **f** for DEGs at N24.

h, Western blot analysis for indicated proteins in WT, *Pten*, *Tsc2* and *Tcf7l1* KO ESCs at N24 after DMSO(-) or Rapamycin(+) treatment. VINCULIN serves as loading control. Representative of n=3 independent experiments.

i, Quantification of p4EBP1 and pS6K by Western blot analysis in WT, *Pten* and *Tsc2* KO cells at N24 after DMSO (light blue) or Rapamycin (dark blue) treatment. Expression was normalised to VINCULIN. Mean and SD for n=3 independent experiments (distinguished by greyscale) are shown. Samples from the same experiment were processed in parallel in the same blot. *p* values from paired, one-tailed t-tests.

j, PCA analysis based on the top 500 DEGs in RNA-Seq data of WT, *Pten* and *Tsc2* KO ESCs at N24 after DMSO (light blue) or Rapamycin (dark blue) treatment.

k, Western blot for indicated proteins in WT, *Pten* and *Tsc2* KO ESCs in 2i, 2i/LIF, N24 and N48. TUBULIN served as loading control.

l, Flow cytometry analysis of Rex1-GFP in WT, *Tcf7l1* and *Pten* KO cells at N48 after CHIRON treatment (CH, green) or without (NT, blue). Representative of n=3 independent experiments.

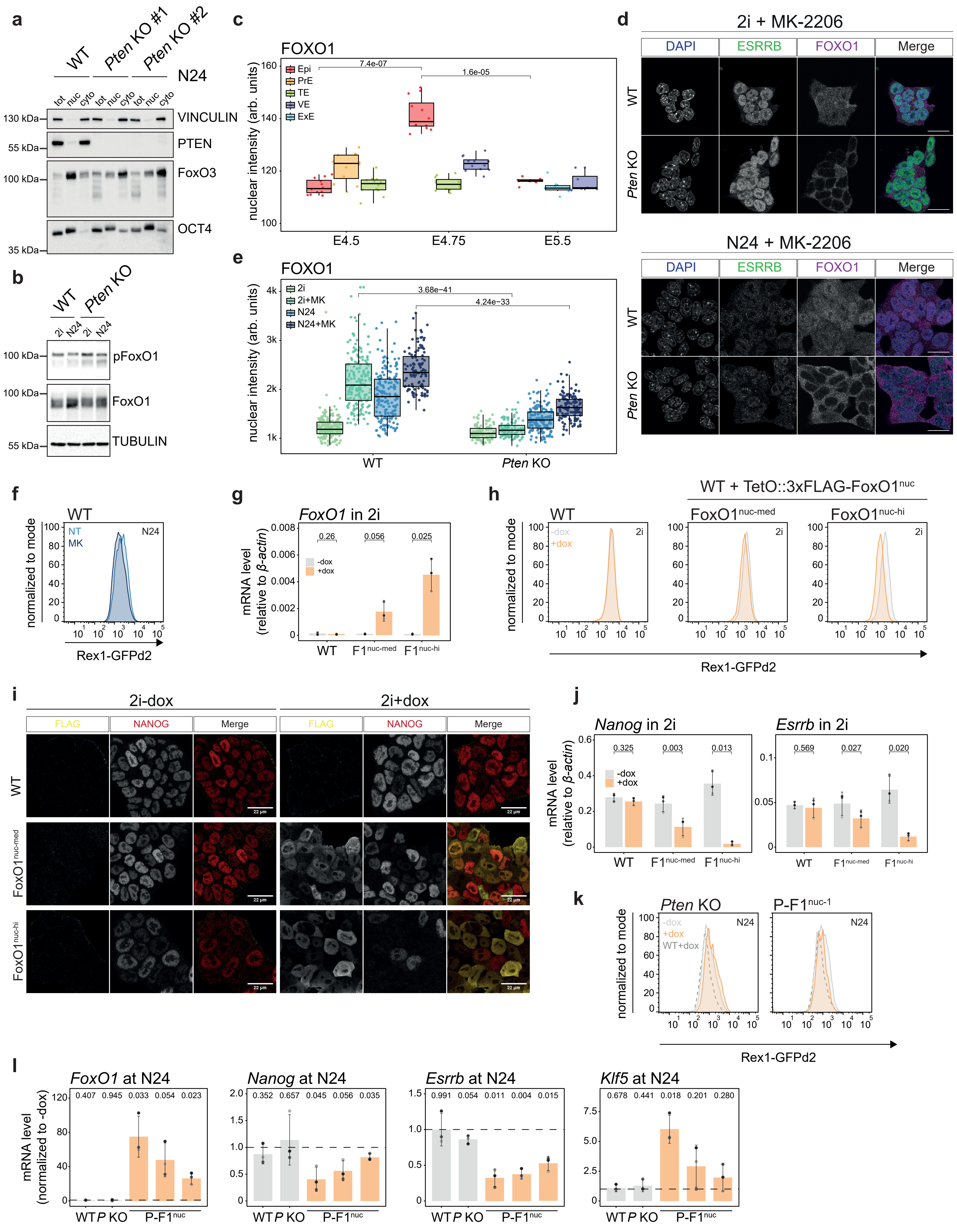

Supplementary Figure 2

Supplementary Fig. 2 | FoxO TFs translocate into the nucleus upon exit from naïve pluripotency.

a, Western blot of nucleo-cytoplasmic fractionation experiments in indicated cell lines at N24. VINCULIN and OCT4 are controls for the cytoplasmic and nuclear fractions, respectively.

b, Western blot for indicated proteins in WT and *Pten* KOs in 2i and N24. TUBULIN served as loading control.

c, Quantification of FOXO1 nuclear intensity (arb. units) measured from confocal images of E4.5 (n=12), E4.75 (n=12) and E5.5 (n=8) embryos. Colours indicate epiblast (Epi), primitive endoderm (PrE), trophectoderm (TE), visceral endoderm (VE), extraembryonic ectoderm (ExE). n=3 independent experiments. *p* values from two-tailed Wilcoxon rank sum tests.

d, Confocal microscopy images after IF showing FOXO1 (purple) and ESRRB (green) in WT and *Pten* KOs in 2i and N24 after MK-2206 treatment. DAPI staining is blue. Representative of n=2 independent experiments. Scale bar=20µM.

e, Quantification of FOXO1 nuclear intensity measured from confocal images of untreated WT (n=189 [2i] and n=164 [N24]), *Pten* KO (n=100 [2i] and n=153 [N24]) and MK-2206 treated cells (WT: n=176 [2i] and n=111 [N24]; *Pten* KO: n=100 [2i] and n=121 [N24]). Representative of n=2 independent experiments. *p* values from two-tailed Wilcoxon rank sum tests.

f, Flow cytometry analysis of Rex1-GFP levels in untreated (NT, light blue) and MK-2206 treated (MK, dark blue) cells at N24. Representative of n=3 independent experiments.

g, *FoxO1* expression levels by RT-qPCR in WT expressing 3xFLAG-FoxO1^{nuc} (FoxO1$^{nuc\text{-}med}$ and FoxO1$^{nuc\text{-}hi}$) in 2i after 8 hours with (+dox, orange) or without (-dox, grey) doxycycline treatment. WT cells are controls. Mean and SD of n=3 independent experiments (distinct shades of grey). Expression normalised to *β-actin*. *p* values from paired, two-tailed t-tests.

h, Flow cytometry of Rex1-GFP levels in WT, FoxO1$^{nuc\text{-}med}$ and FoxO1$^{nuc\text{-}hi}$ cells in 2i after 8 hours with (+dox, orange) or without (-dox, grey) doxycycline. Representative of n=3 independent experiments.

i, Confocal analysis after IF of 3xFLAG-FoxO1 (yellow) and NANOG (red) in WT, FoxO1$^{nuc\text{-}med}$ and FoxO1$^{nuc\text{-}hi}$ cells in 2i with (2i+dox) or without (2i-dox) 8h doxycycline treatment.

j, *Nanog* and *Esrrb* expression by RT-qPCR in WT expressing 3xFLAG-FoxO1^{nuc} (FoxO1$^{nuc\text{-}med}$ and FoxO1$^{nuc\text{-}hi}$) in 2i after 8 hours with (+dox, orange) or without (-dox, grey) doxycycline treatment. n=3 independent experiments (distinct shades of grey). Expression was normalised to *β-actin*. *p* values from paired, two-tailed t-tests.

k, Flow cytometry of Rex1-GFP in *Pten* KOs expressing 3xFLAG-FoxO1^{nuc} (P-FoxO1^{nuc}) at N24 after 8 hours with (+dox, orange) or without (-dox, grey) doxycycline treatment. Rex1-GFP levels of dox-treated WT cells are shown as a grey dashed line. Representative of n=2 independent experiments.

l, Expression of *FoxO1*, *Nanog, Esrrb, Klf4* and *Klf5* by RT-qPCR in *Pten* KO cells expressing 3xFLAG-FoxO1^{nuc} (P-FoxO1^{nuc}) at N24 after 8 hours of doxycycline treatment (orange). WT and *Pten* KO cells are controls (grey). n=3 independent experiments (distinct shades of grey). Expression was normalised to *Rpl32,* and corresponding -dox samples for each cell line were set to one (dashed line). *p* values from paired, two-tailed t-tests.

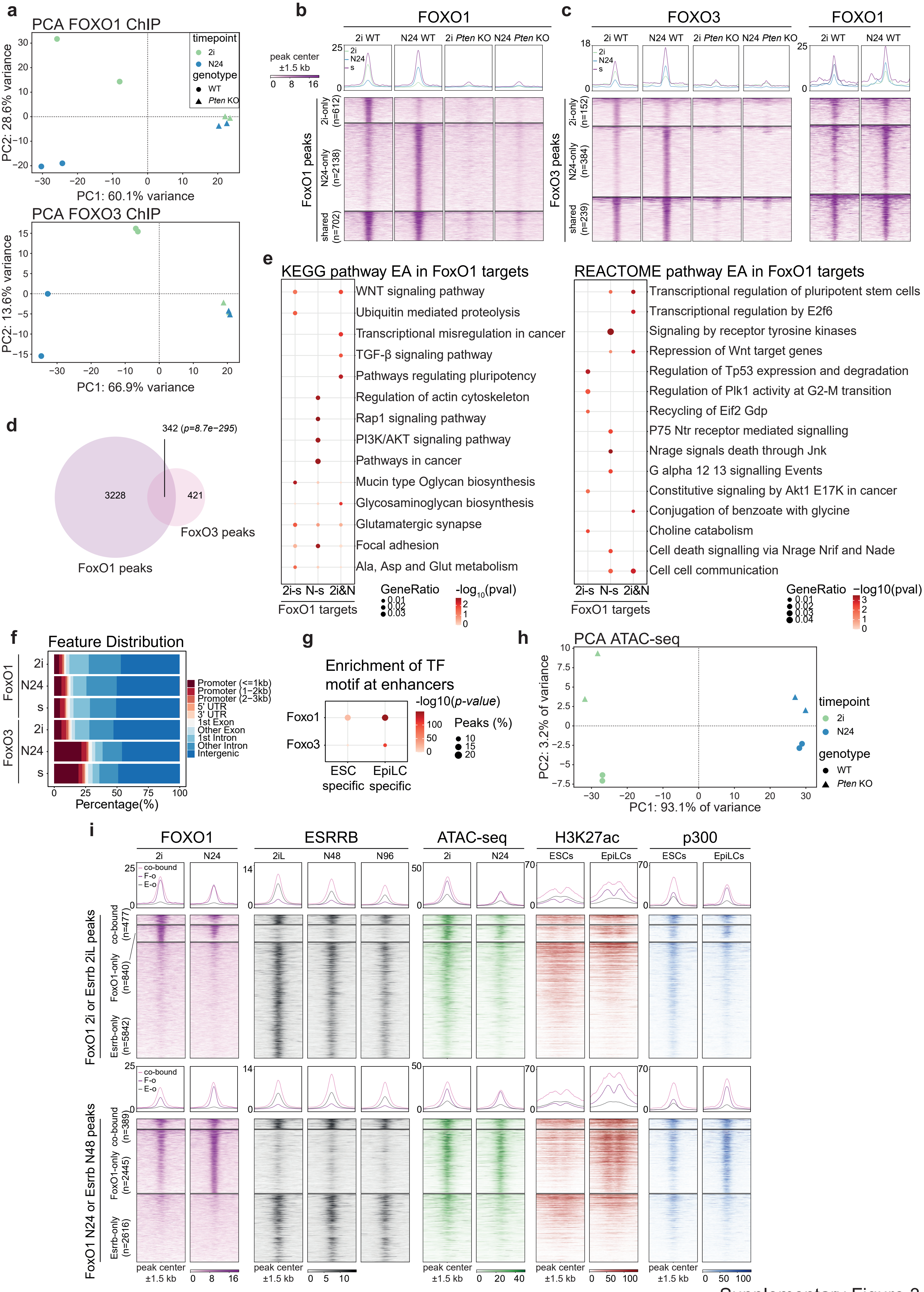

Supplementary Figure 3

Supplementary Fig. 3 | Chromatin dynamics of FoxO TFs at the exit from naïve pluripotency.

a, PCA analysis of FOXO1 (top) or FOXO3 (bottom) ChIP-Seq data in WT and *Pten* KO cells in 2i (green) and at N24 (blue). Each symbol refers to one specific cell line as indicated in the legend.

b, Heatmap showing FOXO1 signal in a 1.5 kb window around the center of FoxO1 peaks, divided in 2i-only peaks (n=612, green), N24-only peaks (n=2138, blue) and shared peaks (n=702, purple), in WT and *Pten* KO cells in 2i or at N24.

c, Heatmap showing FOXO3 signal (left) and FOXO1 signal (right) in a 1.5 kb window around the center of FoxO3 peaks, divided in 2i-only peaks (n=152, green), N24-only peaks (n=384, blue) and shared peaks (n=239, purple), in WT and *Pten* KO cells in 2i or at N24.

d, Venn diagram showing the overlap between FoxO1 (purple) and FoxO3 (pink) peaks. The resulting *p* value from the hypergeometric test of the overlap is shown in italics.

e, KEGG pathway (left) and REACTOME (right) enrichment analysis (EA) on genes associated with indicated FoxO1 peaks (x axis). Top 5 categories enriched in each list are shown on the y axis. Dot colour indicates *p*-values (only $p \leq 0.1$ are shown). Dot size indicates the GeneRatio (ratio between the overlap size and the category size).

f, Feature distribution of FoxO1 and FoxO3 peaks. The categories are indicated in the legend.

g, Enrichment analysis of FOXO1 and FOXO3 motifs in ESC- or EpiLC-specific enhancers. Dot colour and size indicate the *p*-values and the percentage of enhancers ("peaks") with the motif, respectively.

h, PCA analysis of ATAC-Seq data in WT and *Pten* KO cells in 2i (green) and at N24 (blue). Each symbol refers to one specific cell line as indicated in the legend.

i, Heatmaps showing FOXO1, ESRRB, ATAC-seq, H3K27ac and p300 signal in the indicated samples in a 1.5 kb window around the center of top) FoxO1 2i and Esrrb 2iL peaks, divided in co-bound peaks (n=477, pink), FoxO1-only peaks (n=840, purple) and Esrrb-only peaks (n=5842, grey); bottom) FoxO1 N24 and Esrrb N48 peaks, divided in co-bound peaks (n=389, pink), FoxO1-only peaks (n=2445, purple) and Esrrb-only peaks (n=2616, grey).

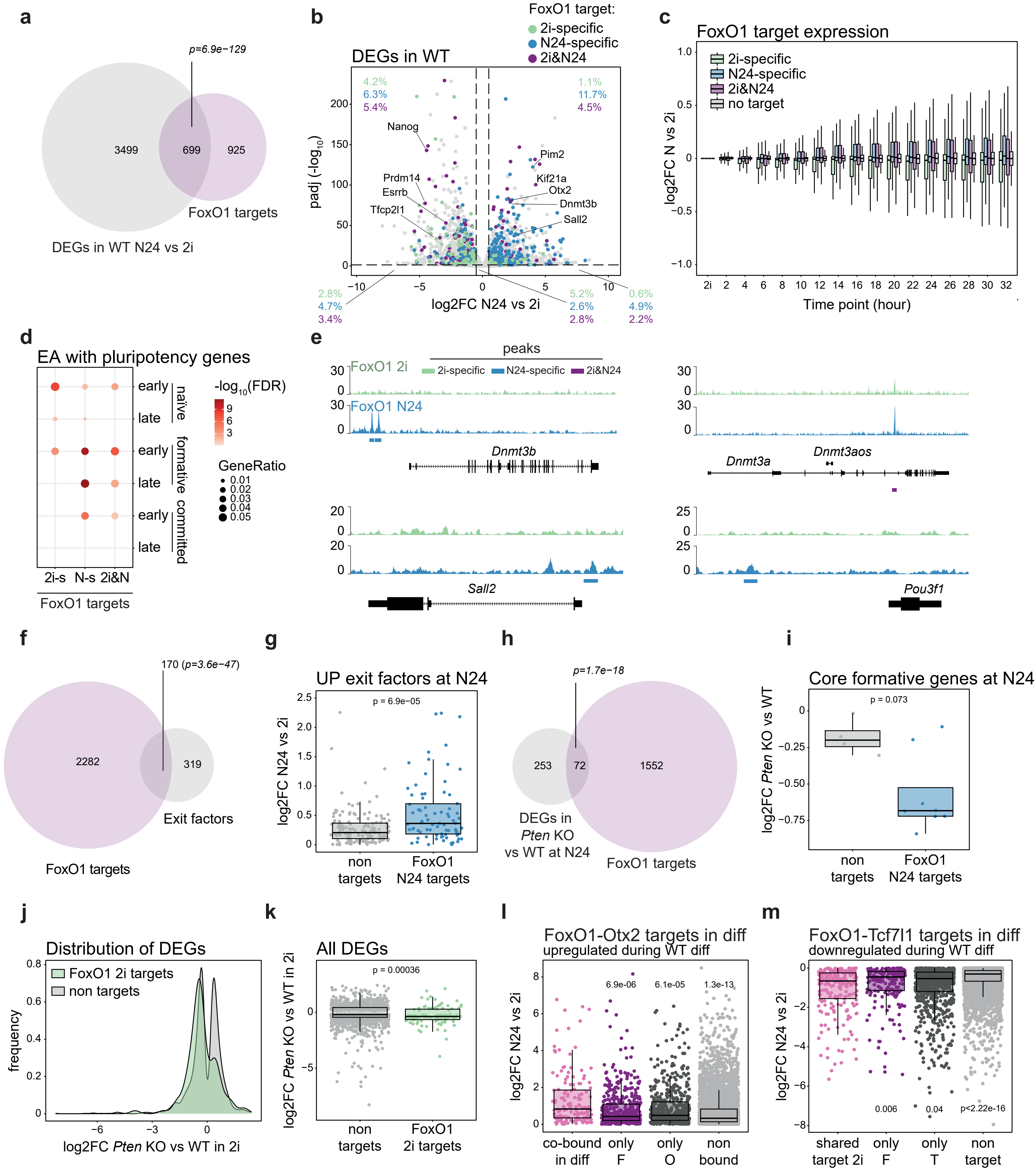

Supplementary Figure 4

Supplementary Fig. 4 | FoxO TF targets are key players in the naïve to formative pluripotency transition.

a, Venn diagram showing the overlap between FoxO1 targets (purple) and DEGs during WT differentiation (|log2FC N24 vs 2i ≥ 0.5, *p-adj.* ≤ 0.05, grey). *p* value from hypergeometric test of the overlap.

b, Volcano plot showing RNA-seq data from WT differentiation. DEGs bound by FoxO1 are colour coded (bound only in 2i [green], only at N24 [blue] or in both conditions [purple]). Selected naïve and formative genes are indicated in the plot. For each quadrant, percentages of FoxO1 2i-only, N24-only and 2i&N24 targets are shown.

c, Boxplot showing expression dynamics of 2i-specific (n=341, green), N24-specific (n=1213, blue) and 2i&N24 (n=611, purple) FoxO1 targets across a 2 hours-resolved WT differentiation time course. Non-targets (n=22387) are grey. Data are shown as log2FC relative to 2i.

d, Enrichment analysis (EA) of FoxO1 targets within sets of naïve, formative and committed genes. Dot-colour indicates the FDR-values (only FDR ≤ 0.05 are shown), dot-size the GeneRatio (overlap size/category size).

e, Genome-browser snapshots showing FoxO1 ChIP-signal in WT on indicated formative markers in 2i (green) and N24 (blue). Called peaks are indicated in the plots as bars (N24-only peaks: blue, shared peaks: purple).

f, Venn diagram showing overlap between FoxO1 targets (purple) and exit factors (grey). *p* value from a hypergeometric test of the overlap.

g, Box plot showing the expression of exit factors upregulated during WT differentiation (n=233, log2FC > 0), divided into FoxO1 N24 targets (blue) or non-targets (grey). Data are shown as log2FC relative to 2i. *p* value from a two-tailed Wilcoxon rank sum test.

h, Venn diagram showing the overlap between FoxO1 targets (purple) and DEGs in *Pten* KO at N24 (|log2FC *Pten* KO vs WT| ≥ 0.5, *p-adj.* ≤ 0.05, grey). *p* value from a hypergeometric test of the overlap.

i, Box plot showing the expression of core formative genes (n=12) in *Pten* KOs at N24, divided into FoxO1 N24 targets (blue) or non-targets (grey) measured by RNA-seq. Data are shown as log2FC relative to WT. *p* value from a two-tailed Wilcoxon rank sum test.

j, Distribution of log2FC of DEGs, (*p-adj.* ≤ 0.05) in *Pten* KOs compared to WT cells in 2i, divided in FoxO1 2i targets (green) and non-targets (grey).

k, Box plot showing the expression of DEGs (n=1655, *p-adj.* ≤ 0.05) in *Pten* KOs in 2i, divided into FoxO1 2i targets (green) or non-targets (grey) measured by RNA-seq. Data are shown as log2FC relative to WT. *p* value from a two-tailed Wilcoxon rank sum test.

l, Box plot showing expression of genes upregulated during WT differentiation (n=7273, lo2FC > 0) that are either co-bound by FOXO1 and OTX2 (pink), bound only by FOXO1 (purple), only by OTX2 (dark grey), or not bound (light grey). Data are shown as log2FC relative to 2i. *p* values from two-tailed Wilcoxon rank sum tests comparing each group to the co-bound group are indicated.

m, Box plot showing expression of genes downregulated during WT differentiation (n=6799, lo2FC < 0) that are either shared targets of FoxO1 and Tcf7l1 (pink), only FoxO1 (purple), only Tcf7l1 (dark grey), or non-targets (light grey) in 2i. Data are shown as log2FC relative to 2i. *p* values from two-tailed Wilcoxon rank sum tests comparing each group to shared target group are indicated.

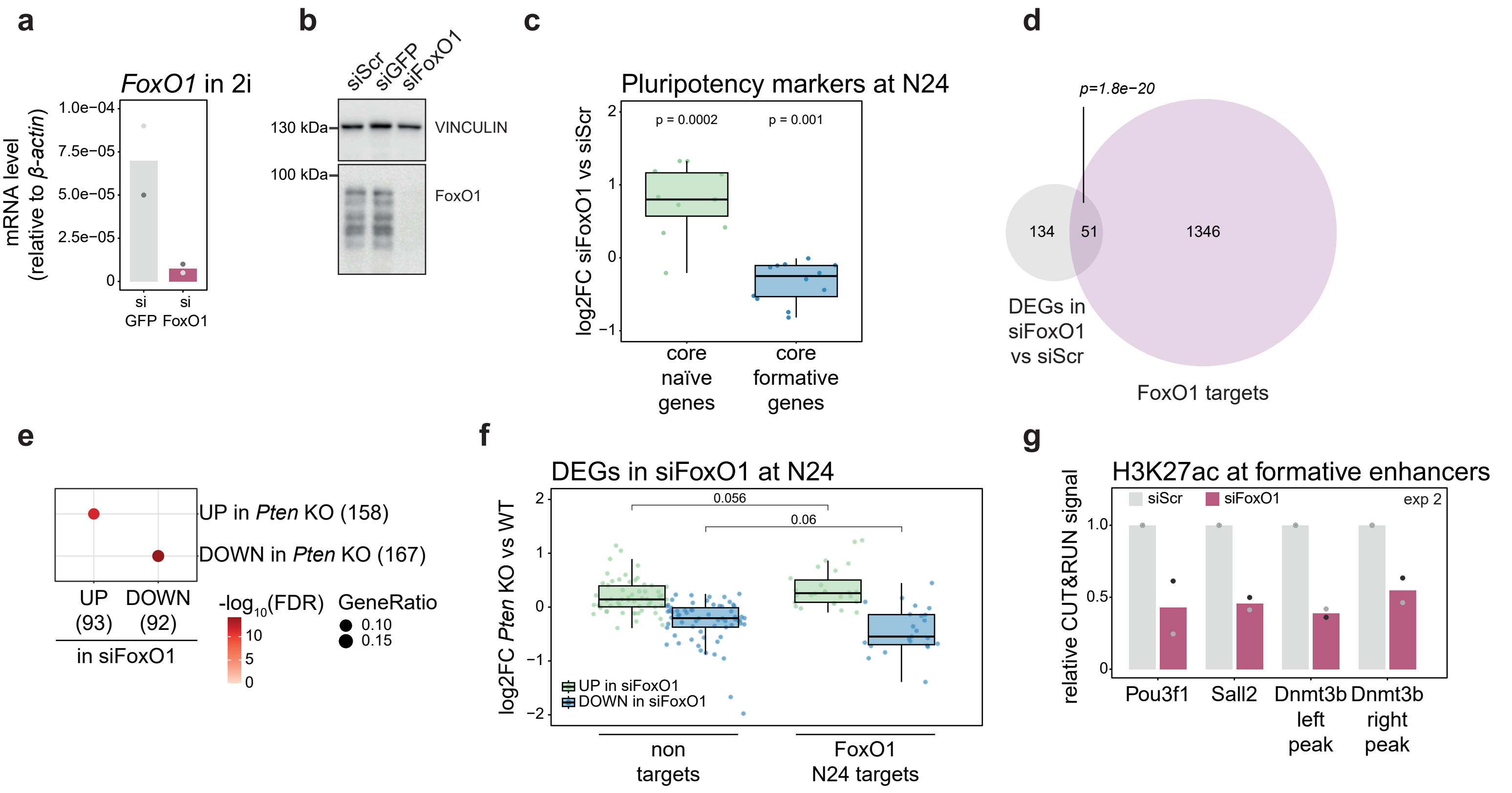

Supplementary Figure 5

Supplementary Fig. 5 | Interference with FoxO1 nuclear shuttling impairs the transition from the naïve to the formative GRN.

a, *FoxO1* expression levels measured by RT-qPCR in WT cells transfected with control (siGFP, grey) or siRNAs targeting FoxO1 (siFoxO1, red). Mean of n=2 independent experiments (depicted as distinct shades of grey) are shown. Expression was normalised to *β-actin*.

b, Western blot analysis for FOXO1 expression in cells transfected with control (siGFP and siScr) or siRNAs targeting FoxO1 (siFoxO1). VINCULIN was used as a loading control. One representative of n=4 independent experiments.

c, Box plot showing the expression of naïve (n=13, green) and formative (n=12, blue) core genes in siFoxO1 at N24 as measured by RNA-seq. Data are shown as log2FC relative to siCtrl. *p* values from two-tailed Wilcoxon signed rank tests.

d, Venn diagram showing the overlap between FoxO1 targets (purple) and genes differentially expressed in siFoxO1 at N24 (DEGs, *p-adj.* ≤ 0.2). *p* value derived from a hypergeometric test of the overlap.

e, Enrichment analysis (EA) of upregulated (UP) and downregulated (DOWN) genes in siFoxO1 at N24 with upregulated (UP) or downregulated (DOWN) genes in *Pten* KO at N24. Dot colour and size indicate FDR values (only FDR ≤ 0.05 are shown) and GeneRatio (overlap size/category size), respectively.

f, Box plot showing expression of up- (n=93, UP, green) or downregulated (n=92, DOWN, blue) genes upon siFoxO1 in *Pten* KOs at N24, divided into FoxO1 N24 targets or non-targets. Data are shown as log2FC relative to WT. *p* value from two-tailed Wilcoxon rank sum test is indicated.

g, CUT&RUN analysis of H3K27ac levels on indicated enhancers after FoxO1 siRNA (red). Signal was normalized to a genomic background region without H3K27ac signal in WT cells. Further, data was normalized to a H3K27ac peak found in *Drosophila* spike-in cells. Data are shown as relative to siScr control (grey). n=2 biological replicates. An independent experiment with n=2 biological replicates is shown in Fig. 5c.

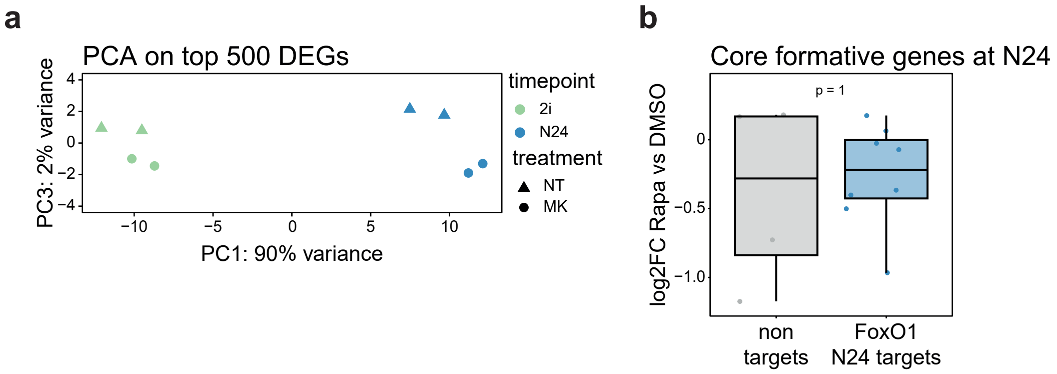

Supplementary Figure 6

Supplementary Fig. 6 | Enforcing nuclear FoxO1 shuttling impairs the transition from the naïve to the formative GRN.

a, PCA analysis based on the top 500 DEGs in RNA-Seq data of WT cells non-treated (NT, triangles) or after treatment with MK-2206 (circles), in 2i (green) or at N24 (blue).

b, Box plot showing the expression of core formative genes (n=12) in WT cells after Rapamycin treatment at N24, divided into FoxO1 N24 targets (blue) or non-targets (grey). Data is shown as log2FC relative to DMSO-treated cells. *p* value from two-tailed Wilcoxon rank sum test is indicated.

Source data for Supplementary Figures 1,2 and 5

Fig. S1a

Chemiluminescence | Colorimetric (PageRuler Plus -Thermo Scientific) | Merge

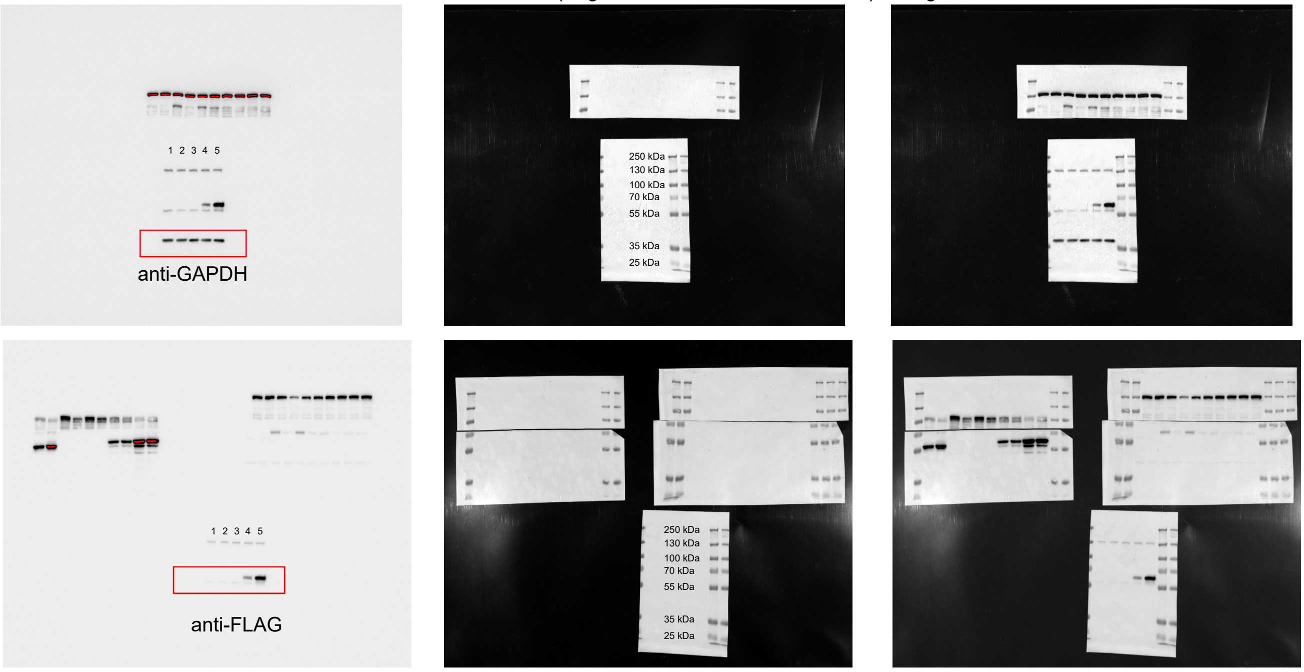

Samples
1 = WT
2 = Pten KO
3 = Pten KO EV
4 = Pten KO FR #1
5 = Pten KO FR #2

Fig. S1d

Chemiluminescence | Colorimetric (PageRuler Plus -Thermo Scientific) | Merge

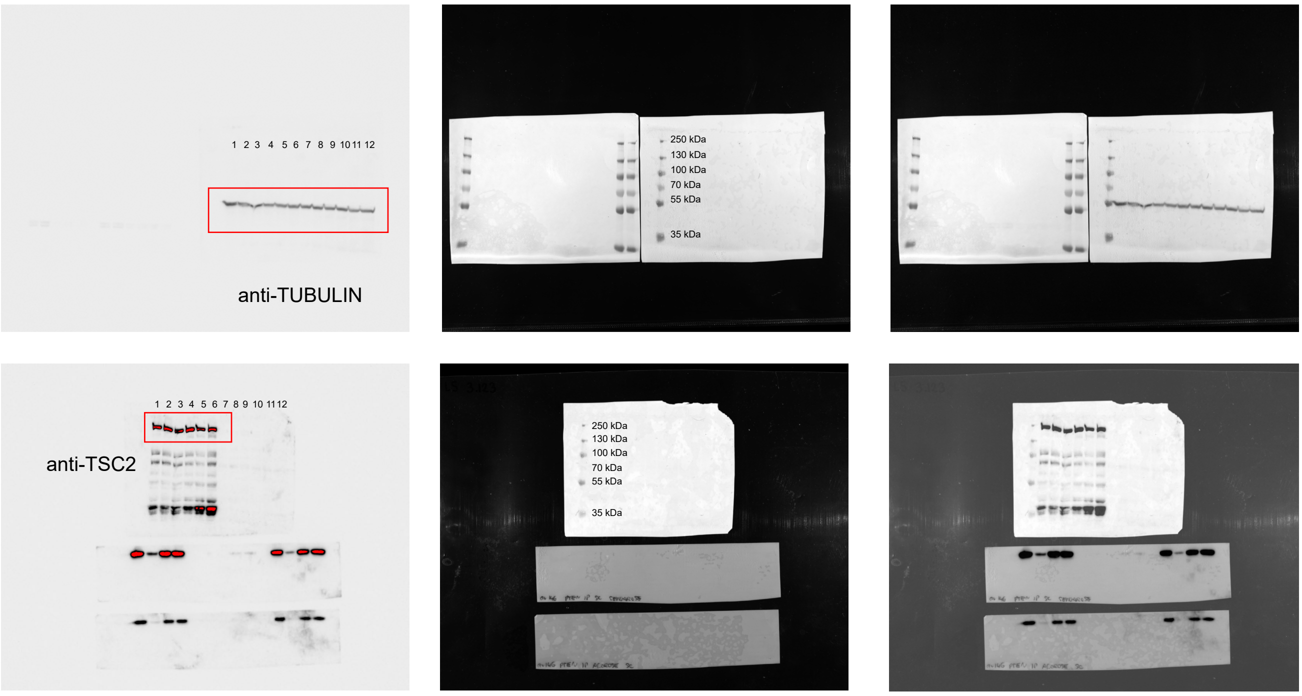

Samples
1 = WT 2i
2 = WT 2iLIF
3 = WT N24
4 = WT N48
5 = WT N72
6 = WT N96
7 = Tsc2 KO 2i
8 = Tsc2 KO 2iLIF
9 = Tsc2 KO N24
10 = Tsc2 KO N48
11 = Tsc2 KO N72
12 = Tsc2 KO N96

Fig. S1h

Chemiluminescence

Colorimetric (PageRuler Plus -Thermo Scientific)

Merge

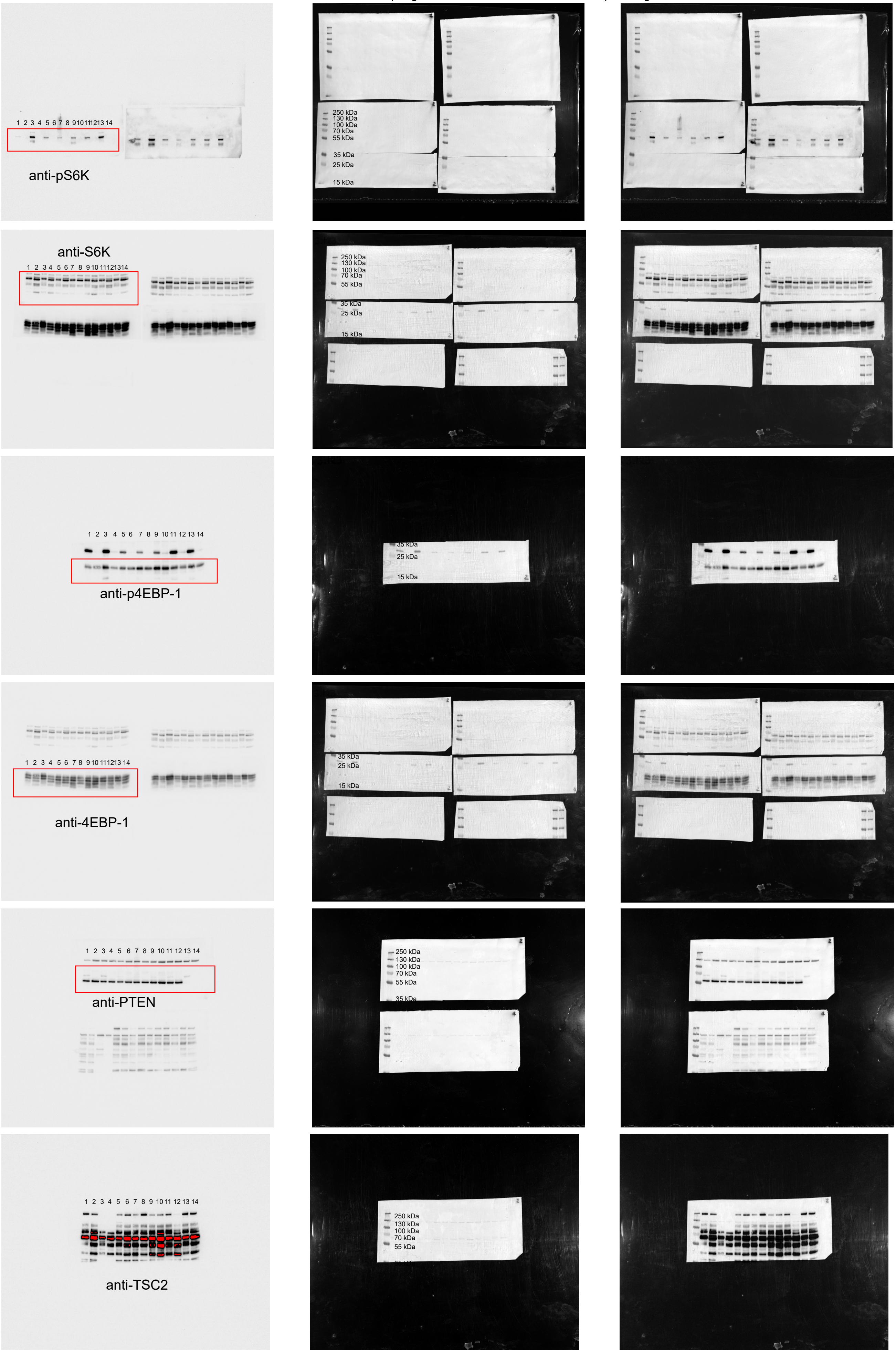

Samples
1 = WT DMSO
2 = WT Rapamycin
3 = Tsc2 KO DMSO
4 = Tsc2 KO Rapamycin
5 = Tcf7l1 KO DMSO
6 = Tcf7l1 KO Rapamycin
13 = Pten KO DMSO
14 = Pten KO Rapamycin

Fig. S1h

Chemiluminescence | Colorimetric (PageRuler Plus -Thermo Scientific) | Merge

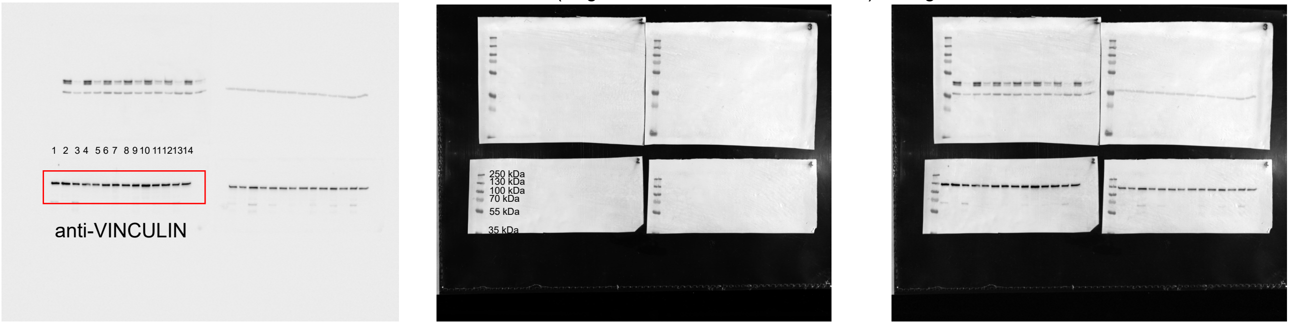

Samples
1 = WT DMSO
2 = WT Rapamycin
3 = Tsc2 KO DMSO
4 = Tsc2 KO Rapamycin
5 = Tcf7l1 KO DMSO
6 = Tcf7l1 KO Rapamycin
13 = Pten KO DMSO
14 = Pten KO Rapamycin

Fig. S1k

Chemiluminescence | Colorimetric (PageRuler Plus -Thermo Scientific) | Merge

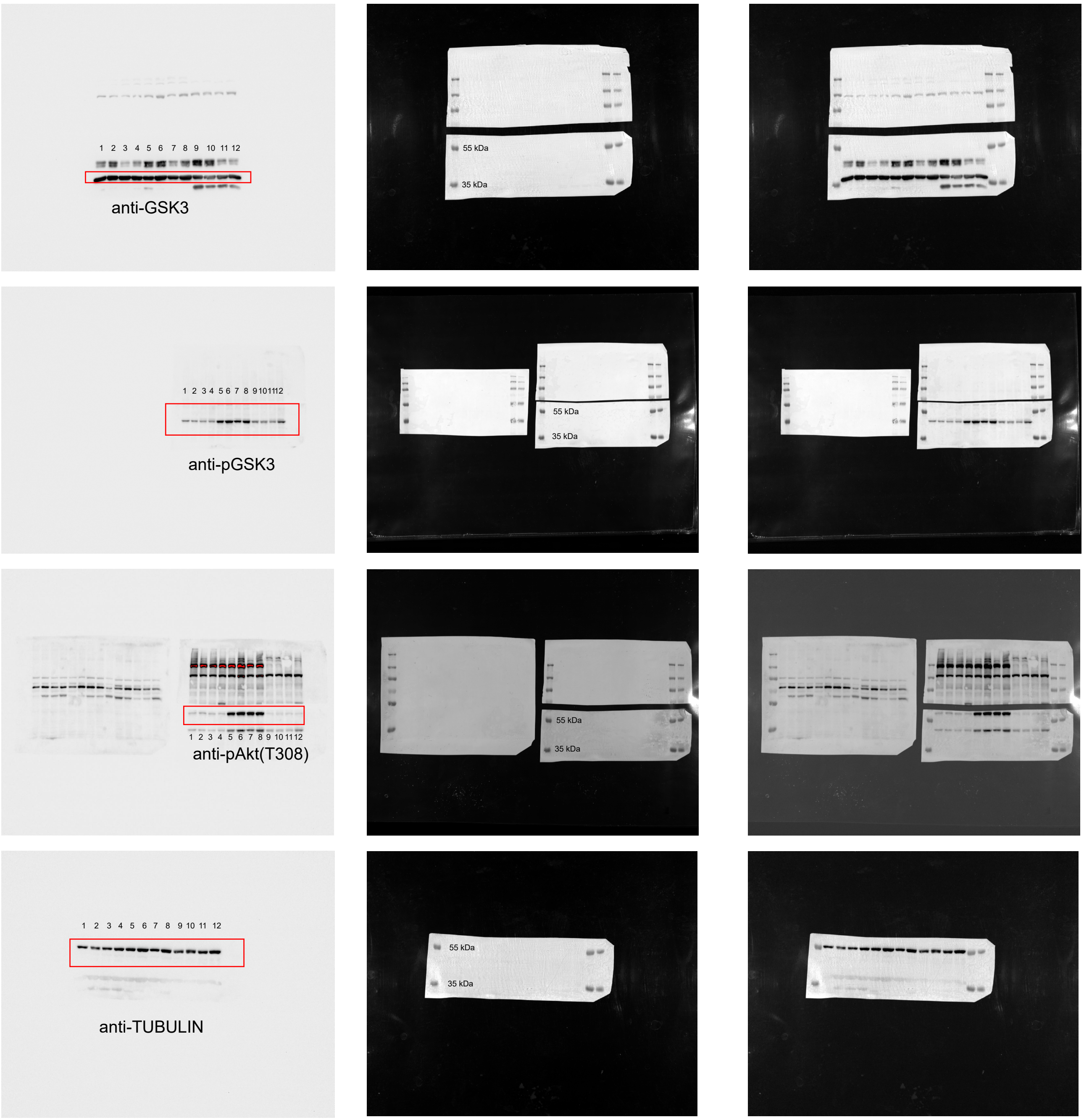

Samples
1 = WT 2iLIF
2 = WT 2i
3 = WT N24
4 = WT N48
5 = Pten KO2iLIF
6 = Pten KO 2i
7 = Pten KO N24
8 = Pten KO N48
9 = Tsc2 KO 2iLIF
10 = Tsc2 KO 2i
11 = Tsc2 KO N24
12 = Tsc2 KO N48

Fig. S2a

Chemiluminescence | Colorimetric (PageRuler Plus -Thermo Scientific) | Merge

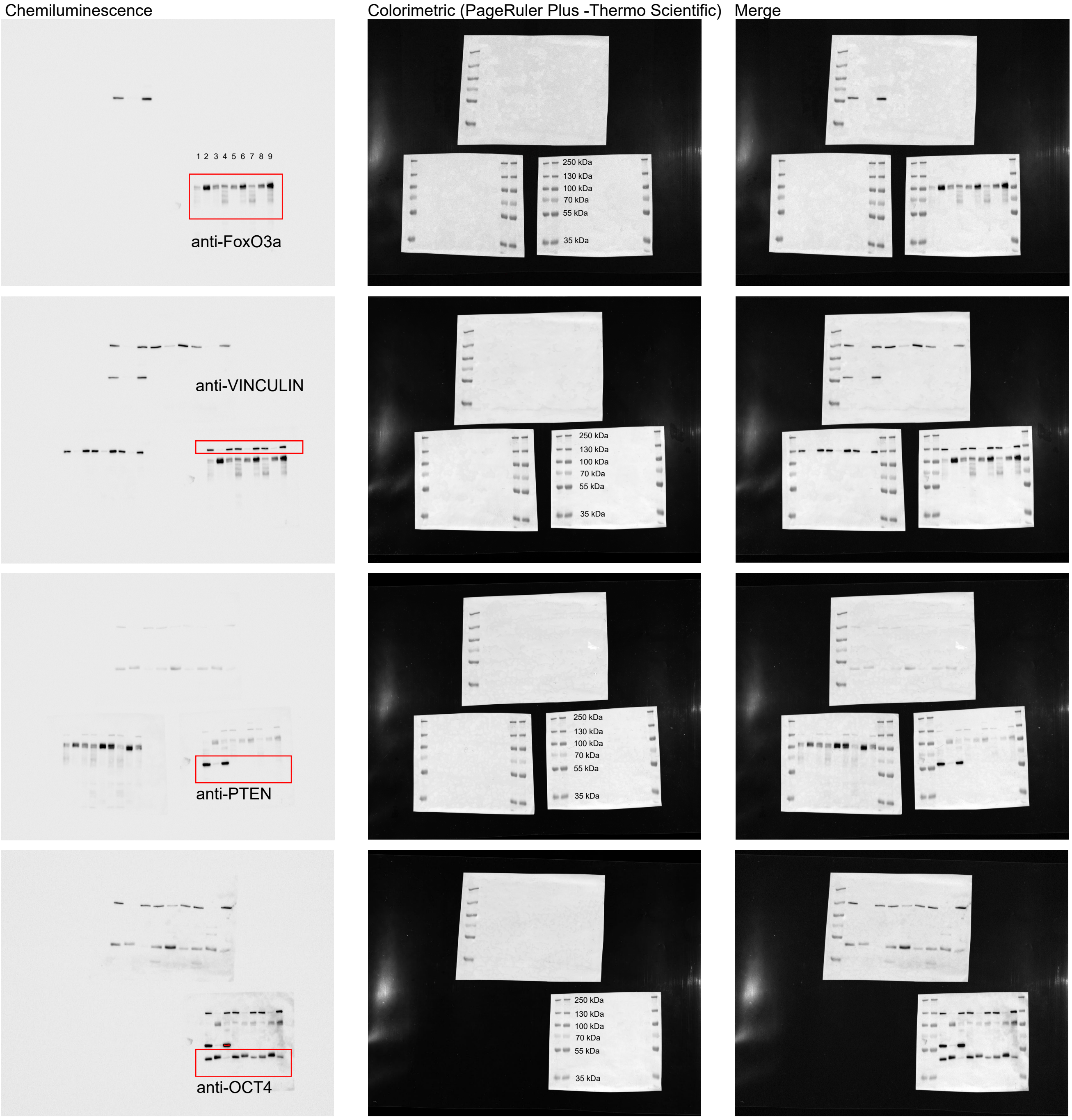

Samples
1 = WT total
2 = WT nuclear
3 = WT cytoplasmic
4 = Pten KO #1 total
5 = Pten KO #1 nuclear
6 = Pten KO #1 cytoplasmic
7 = Pten KO #2 total
8 = Pten KO #2 nuclear
9 = Pten KO #2 cytoplasmic

Fig. S2b

Chemiluminescence | Colorimetric (PageRuler Plus -Thermo Scientific) | Merge

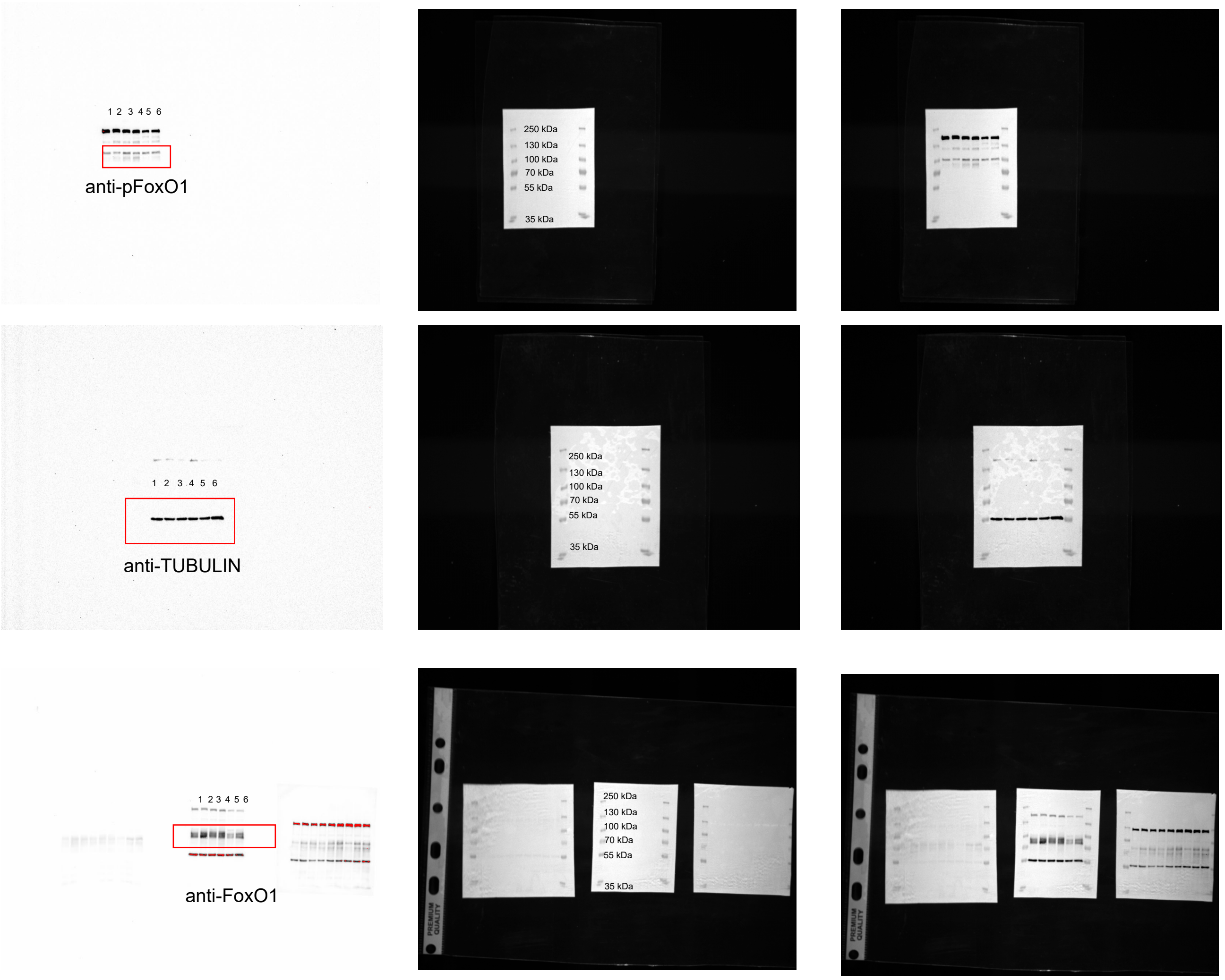

Samples
1 = WT 2i
2 = WT N24
3 = Pten KO 2i
4 = Pten KO N24

Fig. S5b

Chemiluminescence | Colorimetric (PageRuler Plus -Thermo Scientific) | Merge

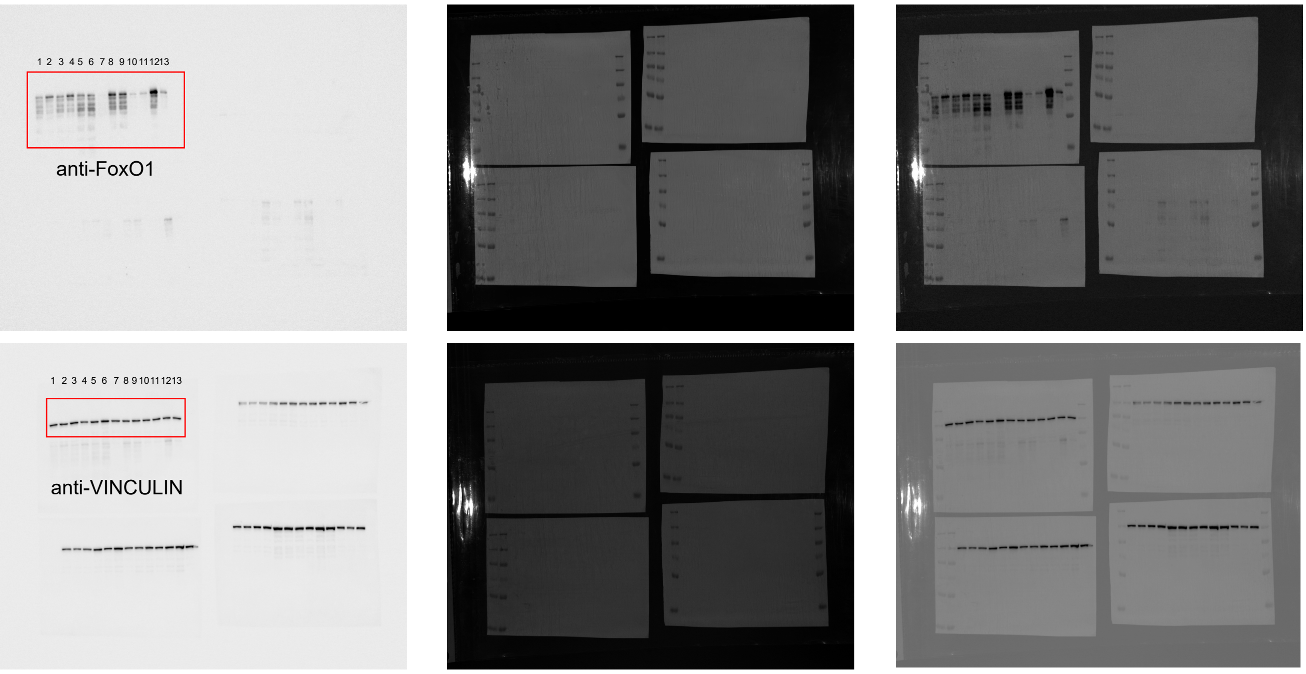

Samples
5 = siScr
6 = siGFP
7 = siFoxO1
