# Supplementary material for: FoxO transcription factors actuate the formative pluripotency specific gene expression programme
Source: Nat Commun. 2024 Sep 9;15:7879. doi: 10.1038/s41467-024-51794-9 (PMC11384738; doi:10.1038/s41467-024-51794-9)
Supplement: Supplementary file 3 — Description of Additional Supplementary Files [file 41467_2024_51794_MOESM3_ESM.pdf]

Description of Additional Supplementary Files

File Name: Supplementary Data 1
Description: Enrichment analysis results

File Name: Supplementary Data 2
Description: Expression data, gene categories and genomic locations

File Name: Supplementary Data 3
Description: Primers used in this study
