# Supplementary figures and images for: FoxO transcription factors actuate the formative pluripotency specific gene expression programme
Source: Nat Commun. 2024 Sep 9;15:7879. doi: 10.1038/s41467-024-51794-9 (PMC11384738; doi:10.1038/s41467-024-51794-9)

Fig. 1d top part

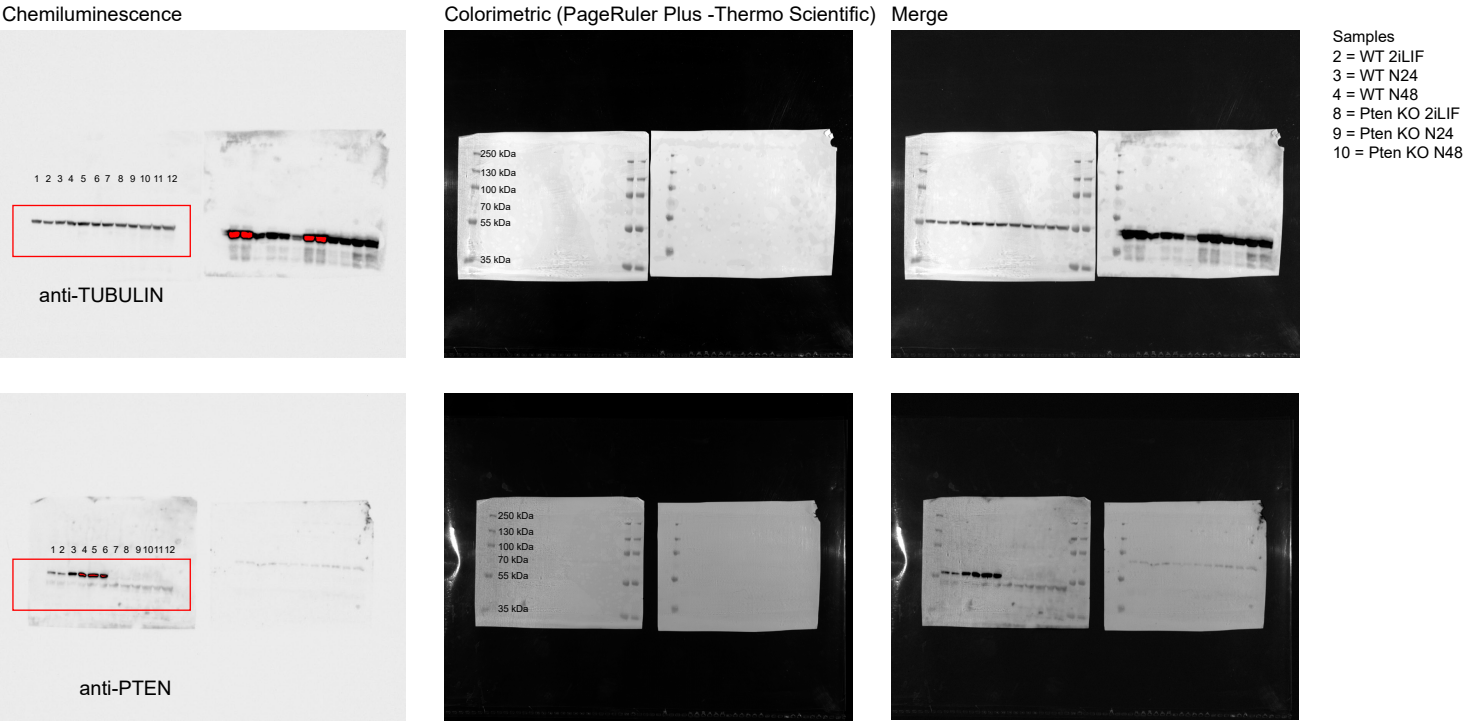

Fig. 1d bottom part

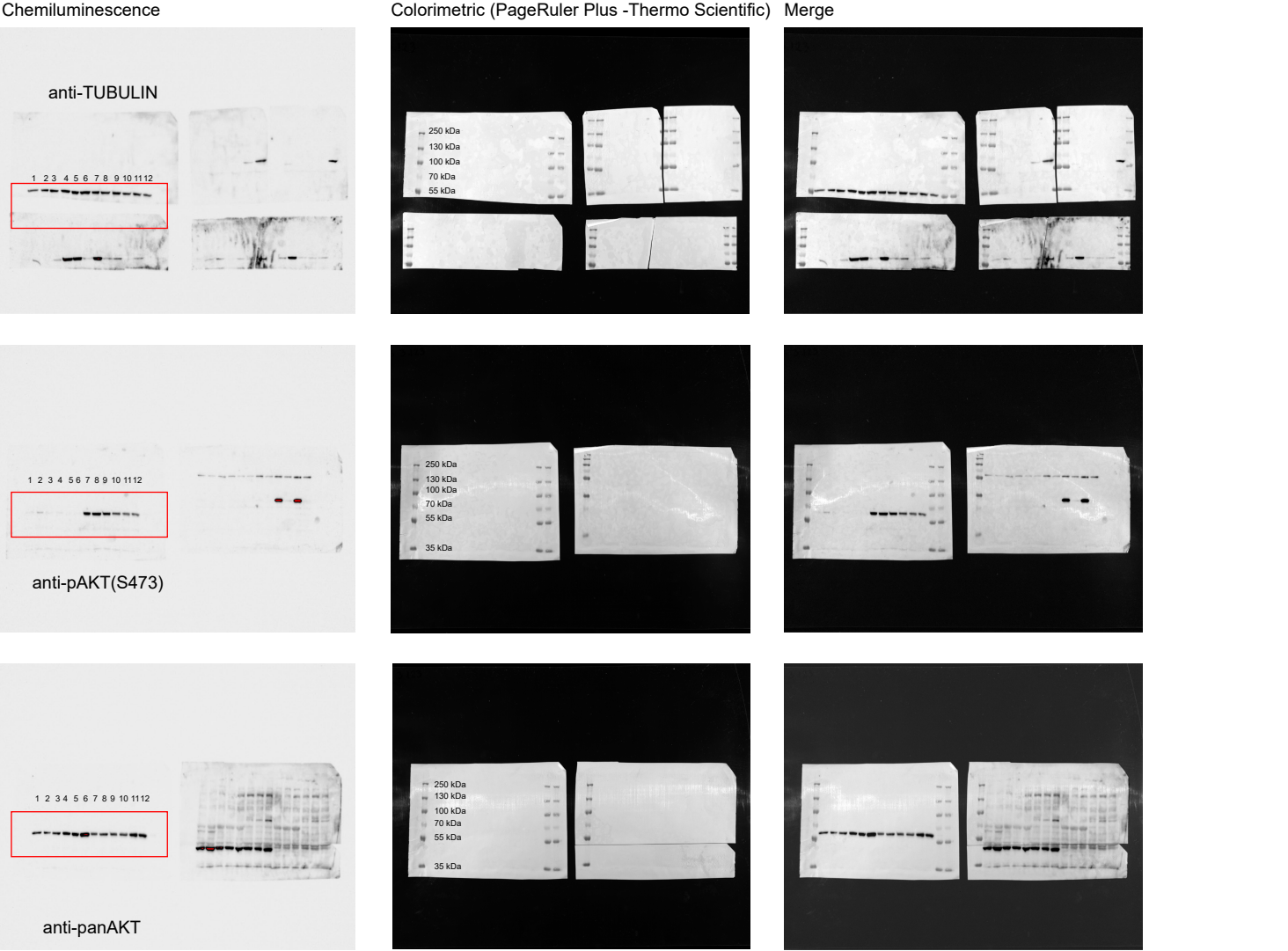

Supplement: Supplementary file 8 — Source Data [file 41467_2024_51794_MOESM8_ESM.zip › Source Data Fig1d.pdf]
